# Supported Employment Programmes for People With Psychosis or Schizophrenia – Full Cycle Audit

**DOI:** 10.1192/bjo.2024.584

**Published:** 2024-08-01

**Authors:** Mounika Iderapalli, Mohamed Ibrahim, Shaimaa Aboelenien

**Affiliations:** Essex Partnership University Trust, Rochford, United Kingdom

## Abstract

**Aims:**

Our aim was to see if the following have been done during medical reviews:

1. For service users with a diagnosis of psychosis or schizophrenia; is there clear documentation of employment status in case notes?
2. To see if supported employment/alternative education or occupational activity is being offered to unemployed service users in their Outpatient Clinic Appointments.
3. Whether acceptance or refusal of offered support is being documented and followed up.

This is a re-audit and it was done to check the compliance of our service with recommended NICE guidelines following the first cycle audit.

The NICE Guidelines (CG178 – Psychosis and Schizophrenia in Adults: Prevention and Management and NG181 – Rehabilitation for adults with complex psychosis) recommend the following for all patients with diagnosis of psychosis or schizophrenia.

Standard 1:

Offer supported employment to people with psychosis or schizophrenia who wish to find or return to work *(CG178 Psychosis and schizophrenia in adults: prevention and management – 1.5.8 – Employment, Education & Occupational Activities)*.

Standard 2:

Facilitate alternate educational or occupational activities for people who do not wish to pursue mainstream education or work *(NG181 Rehabilitation for adults with complex psychosis – 1.8.9 Engagement in community activities, including leisure, education and work).*

**Methods:**

This re-audit was carried out in the Community Adult Mental Health Services at Taylor Centre, Southend.

A list of service users that attended Outpatient Services at the Taylor Centre during the months of August and September 2023 was obtained.

Case notes of service users with diagnosis of psychosis or schizophrenia that attended an Outpatient Clinic over the 2-month period as new appointment or follow up were reviewed retrospectively. The 2 months (August and September) were chosen at random to achieve a reasonable sample size.

Service users within age range of 18–68 years were selected as they fall into the working age group range in UK.

The following details were checked:

1. Is the service user's employment status recorded in case notes?
2. For those who are unemployed – is supported employment offered?
3. Service User's acceptance or refusal and any alternative educational or occupational activity facilitated if they refuse mainstream work.

The data was collected on an Excel spreadsheet and analysed.

**Results:**

Findings:

- In the case notes of our sample, Employment status was documents in 97% of the cases; 78% were not actively seeking work.
- The type of appointment in which employment was most often discussed was in the follow up appointments = 87% of the cases.
- Supported Employment was offered to 38% of the sample. It was noted that 64% of service users that were offered supported employment had declined the offer.

Based on individual service users’ circumstances alternative educational or employment options like prevocational training was offered to 47% of the sample. This is a notable improvement from 26% in first cycle.

**Conclusion:**

There was good documentation of employment status in the case notes of 97% of the sample which shows that Employment history is being taken for almost all the service users that attend our Outpatient Service.

The type of appointment in which employment was most often discussed was in the follow up appointment (87% of the cases). This could be due to service users’ mental state at the time of their first appointment, with them being unwell; hence it may be unsuitable to discuss employment options at that time.

Supported Employment was offered to 38% of the sample. The initial audit showed that this was offered to 35% of the non-working sample; therefore, a small improvement in offering supported employment has been noted. However, it is worth noting that 64% of the people that were offered supported employment have declined the offer, which reiterates the attitude towards mainstream employment in people with serious mental illness.

Only 33% of the service users were followed up, but this could be due to the fact that some of the service users are still waiting to be seen in clinic.

Overall, there has been an improvement following the initial audit especially in offering other educational activities if supported employment is not appropriate or if the service user is not interested.

